# Gestational weight gain and offspring’s cognitive skills: a systematic review and meta-analysis

**DOI:** 10.1186/s12887-020-02429-7

**Published:** 2020-11-26

**Authors:** Jose Alberto Martínez-Hortelano, Celia Álvarez-Bueno, Iván Cavero-Redondo, Ángel Herráiz-Adillo, Carlos Berlanga-Macías, Vicente Martínez-Vizcaíno

**Affiliations:** 1 Universidad de Castilla-La Mancha, Centro de Estudios Sociosanitarios, Cuenca, Spain; 2 Universidad Politécnica y Artística del Paraguay, Asunción, Paraguay; 3 Universidad Autónoma de Chile, Facultad de Ciencias de la Salud, Talca, Chile

**Keywords:** Pregnancy, Gestational, Weight gain, Cognition, Children

## Abstract

**Background:**

Gestational weight gain has been associated with some adverse perinatal outcomes, but few studies have examined the association between gestational weight gain and offspring’s cognition and their conclusions are inconsistent. Our systematic review and meta-analysis aimed to synthesize the evidence regarding the association between gestational weight gain and offspring’s cognitive skills.

**Methods:**

In this systematic review and meta-analysis (PROSPERO number, CRD42017073266), we systematically searched MEDLINE, EMBASE, Web of Science and the Cochrane Library for studies examining association between gestational weight gain and offspring’s cognitive skills, without restriction in study design or language. Two reviewers extracted in an independent way the data. The Quality of Reporting of Observational Longitudinal Research scale was used to assess the quality of included studies. Effect size (ES) for adjusted models and their corresponding 95% confidence intervals were calculated for (i) intelligence quotient, (ii) language related skills and (iii) mathematic related skills comparing offspring’s cognitive skills when gestational weight gain was within recommendations (as reference) with those from mothers whose gestational weight gain was above or below the recommendations.

**Results:**

Thirteen studies were included. There was a positive trend that associated gestational weight gain above recommendations with better offspring’s intelligence quotient, although not statistically significant (ES 0.02, 95% CI -0.00, 0.05; I^2^ = 0.00%).

**Conclusions:**

There is a not significant positive association between gestational weight gain above recommendations and intelligence quotient and some studies reported associations between gestational weight gain and offspring’s cognitive skills. Our analyses confirm a wide variability in the results of studies published so far and highlights the need for conducting studies including specific samples of pregnant women by pre-pregnancy body mass index and trimester of pregnancy.

**Supplementary Information:**

The online version contains supplementary material available at 10.1186/s12887-020-02429-7.

## Background

Recommendations for gestational weight gain (GWG) have been a debatable issue during the last three decades. In 2009, the Institute of Medicine (IOM) updated GWG recommendations published in 1990 by The National Academy of Science [1], changing pre-gestational body mass index (BMI) classification and suggesting less GWG for obese women to improve perinatal outcomes [2]. Nowadays, according to the IOM recommendations in developed countries, 42.9 and 20% of pregnant women had excessive or insufficient GWG, respectively, making it a growing public health problem [3–5].

Excessive GWG increases the risk of some adverse perinatal outcomes, such as caesarean delivery, hypertensive disorders of pregnancy, macrosomia, neonatal hypoglycaemia or shoulder dystocia [6]. Furthermore, excessive GWG has been associated with long-term effects, such as mother’s postpartum weight retention [7]; and higher risk of childhood obesity [8] or neurodevelopmental impairment in children [9]. Conversely, insufficient GWG has been related with preterm birth [6].

Offspring’ cognitive skills have been related to mental and physical health [10, 11], and some observational studies have reported positive, negative or null association between GWG as exposure and neurodevelopment in childhood and adolescence [9, 12–17]. This fact could be due offspring’s neurodevelopment is associated with several factors throughout the different stages of life [18]. During antenatal period, folic acid supplementation could have a positive effect on cognitive development, [19] as well as the treatment with magnesium sulfate or corticosteroids in preterm births that could prevent neurodevelopmental delay. Furthermore, breastfeeding [20, 21], physical activity, [22] education [23] and a good home environment [24, 25] could improve cognitive development. However, iron deficiency during childhood, [18, 26] lower maternal cognition test scores or low socioeconomic status have been associated with worse cognitive development [25].

Considering the potential effect of GWG on offspring’s cognition, it seems necessary to examine the evidence on the association between GWG and children’s cognitive development. Thus, our systematic review and meta-analysis aim to synthesize the evidence regarding the association between GWG and offspring’s cognitive skills, distinguishing among intelligence quotient (IQ), language and mathematics-related skills.

## Methods

This systematic review and meta-analysis was reported according to the Cochrane Collaboration Handbook recommendations [27] and Preferred Reporting Items for Systematics Reviews and Meta-Analysis PRISMA [28]. It has been registered in PROSPERO (Registration number: CRD42017073266).

### Search strategy

**Fig. 1:**
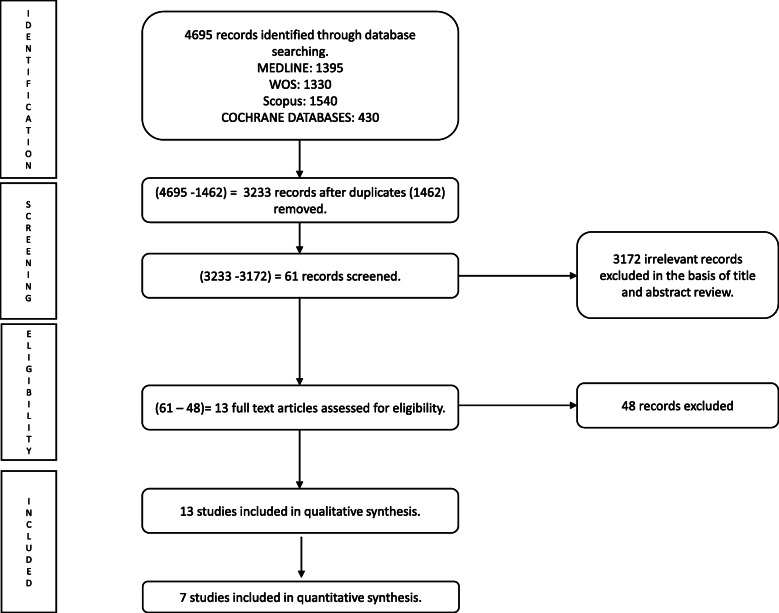
Preferred Reporting Items for Systematic Reviews and Meta-Analyses flow diagram of identification, screening, eligibility and inclusion of studies

Studies were identified through the following databases: MEDLINE, Web of Science, Scopus and Cochrane Library, from their inception to April 2020 without language restriction (Fig. 1). The search strategy included a combination of the following terms: (i) population: “pregnancy”, “gestational maternal”, “antepartum”, “prenatal”; (ii) exposure: “weight gain”, “weight change”, “obesity”; (iii) cognitive skills: “academic achievement”, “academic grades”, “academic behaviour”, “academic behavior”, “academic performance”, “academic”, “attention”, “classroom behaviour”, “classroom behavior”, cognition, “cognitive development”, “cognitive function”, “cognitive control”, “cognitive achievement”, “executive”, “executive function”, “intellectual”, “intelligence”, “neurodevelopment”, “memory”, “metacognition”; (iv) population: “birth”, “infant”, “child”, “childhood”, “children”, “offspring”, “adolescence” (Table S1). The search was manually managed for each database and the references of the selected studies were reviewed to identify additional studies.

### Inclusion criteria

We included studies examining the association between GWG and offspring’s cognitive skills if they met the following inclusion criteria: (i) outcomes: offspring’s cognition assessed by standardized test scores or academic achievement; (ii) study design: observational or experimental studies; (iii) exposure: GWG; (iv) participants: mothers and their offspring without age restriction. Studies were excluded when they were focused exclusively on: (i) mothers with intellectual disabilities; (ii) mothers with diabetes, preeclampsia or cardiovascular problems; (iii) offspring with developmental disorders/diagnoses like symptoms of autism spectrum disorder, because studies in which all children are affected with any detected delay in communication, adaptation, cognition or socio-emotional domains could modify the effect of the main exposure, GWG, on offspring’s cognition and therefore might bias the estimates of meta-analysis.; and (iv) new-borns not born at full term (between 37 to 41 weeks of gestation).

The literature search was performed independently by two reviewers (J.A.M.H. and C.A.B.) and disagreements were solved by consensus. Both reviewers are health professionals and have extensive experience in systematic reviews and meta-analysis. The two reviewers independently examined the titles and abstracts of the studies. Full articles of all studies that appeared to fulfil the inclusion criteria or where there was some uncertainty were obtained for the two reviewers to independently assess their eligibility. Studies that did not fulfil the selection criteria were excluded. The reviewers checked the included and excluded studies and verified the reasons for inclusion/exclusion. Any discrepancies were resolved by consensus and arbitration by a third reviewer (V.M.V.) that is health professional and has extensive experience in systematic reviews and meta-analysis. Reasons for exclusion were coded for both the initial assessment phase and the review phase of full text articles. The PRISMA flowchart will be used to document the study selection process. Kappa interrater agreement was 0.90.

### Data extraction and quality reviews process

The following data were extracted from the original reports: (i) study data (author, year of publication, country, year of birth, sample size of mothers and offspring, GWG classification criteria); (ii) characteristics of participants (offspring’s age at evaluation and maternal variables: age at delivery, pre-pregnancy BMI and GWG); (iii) tools and/or scales used for assessing offspring’s cognitive skills, (iv) cognition domains evaluated and (v) adjustment variables. Duplicated publications were identified based on the data extracted, when multiple articles from a study were identified on the same exposure and outcome variables, only the report with the largest sample size was included; However, if the study samples were independent or they reported different cognitive skills, they were treated as separate studies. If we needed additional information or clarification about any study, authors were contacted.

Data extraction was independently performed by two reviewers (J.A.M.H. and C.A.B.), and inconsistencies were solved by consensus. A third researcher was asked when consensus was not reached (V.M.V.).

Since no experimental study was retrieved, the Quality of Reporting of Observational Longitudinal Research scale was used to evaluate the risk of bias [29]. This rating list consists of 33 items and each criterion was assessed as “yes” (=1), “no” (=0) or “not applicable” (=?), resulting in a total score that ranges from 0 to 33.

Quality assessment was independently performed by two reviewers (J.A.M.H. and C.A.B.), and inconsistencies were solved by consensus. A third researcher was asked when consensus was not reached (V.M.V.).

### Analysis

Effect size (ES) and their respective 95% confidence intervals (95% CI) were used to examine the association between excessive or insufficient GWG and offspring’s cognition. We considered three cognitive domains: IQ, language-related skills and mathematics-related skills. A standardized mean difference score was calculated for each pre-pregnancy weight status category as an estimate of ES [30]. When studies provided a linear regression β coefficient, it was used to calculate a standardized mean difference score [30, 31]. If a study reported stratified data, we performed a pooled estimation to unify data, for example when a study provided data by trimester of pregnancy, we combined data to obtain pooled results throughout pregnancy. The Mantel-Haenszel fixed-effect method [32] was used to compute pooled ES estimates and their respective 95% CI, which were used to examine the association between GWG above or below recommendations and offspring’s cognition, using GWG within recommendations as reference. In the case that one study reported more than one assessment of the same cognition domain at different ages we pooled them; we also polled the estimates of the same domain of cognition measured using different instruments. In the forest plots used to depict the ES of each study and the pooled ES estimates, a negative ES value indicated lower cognitive skills scores in offspring whose mothers had not recommended GWG as compared with offspring whose mothers had GWG within recommendations. The heterogeneity of results across studies was assessed by I^2^ statistic and it was classified as: not important (0 to 30%); moderate (30 to 50%); substantial (50 to 75%) and considerable (75 to 100%), also the corresponding *p*-values were considered [33].

For the analyses, we used: (i) the most adjusted models reported by included studies; (ii) GWG classification criteria published in 1990 by The National Academy of Science or own criteria stablished by original articles were considered similar to those of 2009 IOM recommendations. Furthermore, we conducted subgroup analyses by GWG classification (one group with 2009 IOM’s guidelines and other group with the rest classifications) and by age (stratified as pre-schoolers, from 2 to 6 years old, and schoolers, between 6 and 12 years old).

Sensitivity analyses was conducted by removing studies one by one in order to evaluate the robustness of the summary estimates.

Finally, publication bias was evaluated by visual inspection of funnel plot, and according to the method proposed by Egger [34]. Statistical analyses were performed using StataSE 15.

## Results

From 4498 articles retrieved through the systematic search, 61 were eligible for full-text assessment. Finally, 13 studies were included in the qualitative synthesis [9, 12–17, 35–40] and seven in the quantitative synthesis (Fig. 1) [13, 14, 17, 35, 38–40].

We only included 7 studies in quantitative synthesis for some reasons [13, 14, 17, 35, 38–40]: one study did not provide a reference group of GWG to compare the association between GWG and offspring’s cognition [12]; three did not provide quantitative data of offspring’s cognitive skills that we studied [9, 16, 36]; and three studies shared the same sample and we only included them in the same pooled when assessed different domain of cognition [9, 12, 14].

### Study characteristics

Table 1 summarizes the main characteristics of the included studies. Included studies were published between 1981 and 2018. Ten studies were conducted in the United States [9, 12, 14, 16, 17, 35–37, 39, 40], one in Norway and Sweden [13], United Kingdom [38] and China [15]. All are cohort studies: eight studies reported data for one follow-up evaluation [9, 13, 15–17, 36, 37, 40], two studies for two follow-up evaluations [12, 14, 39] and two studies provided data for three follow-up evaluations [35, 38].

**Table 1:** Characteristics of studies included in the systematic review and meta-analysis

Study	Country	Studu design	Years of recruitment	n mothers	Mother’s age (i)	GWG classification	n offspring	Offspring’s age (i)	Measuring tools and cognitive domains	Adjustement variables
Gage et al. 2013 [38]	UK	Cohort	1991–1992	8653	28.52 (4.75)	IOM 2009	5836 5191 7339	4 8 16	School entry assessment score. Wechsler Intelligence Scale for Children: - → Standarized IQ. School scores.	**Maternal’s variables**: age, gestational age, parity, education, pregestational BMI, tobacco consumption and type of delivery. **Offspring’s variables**: sex, age at evaluation.
Hinkle et al. 2016 [13]	Norway and Sweden	Cohort	1986–1988	344	29.1 (4.2)	IOM 2009	552	5.2 (0.2)	Wechsler Preschoolers and Primary Scales of Intelligence (WPPSI-R), Full Scale: - → Verbal IQ. - → Performance IQ. - → Full scale IQ.	**Maternal’s variables**: age, parity, education, IQ, socioeconomic status, civil status, pregestational BIM, prepregnancy diseases, smoking and alcohol use, nausea and vomiting during pregnancy. **Offspring’s variables**: sex, age at IQ assessment. Sensitive analysis limited to full-term birth.
Huang et al. 2014 [9]	USA	Cohort	1959–1976	29,942	21.3 (8.4)	IOM 2009	8059 children with siblings. 22,513 children with no siblings.	7.1 (0.8)	Stanford–Binet intelligence scale and Graham–Ernhart Block Sort test. Wechsler Intelligence Scale for Children (WISC I): - → Verbal IQ - → Performance IQ - → Full scale IQ.	**Maternal’s variables:** age, race, education, civil status, socioeconomic status, parity, smoking in pregnancy and hospital.
Keim et al. 2012 [14]	USA	Cohort	1959–1973	31,968	< 20: 20.3% 20–29: 64.4% 30–34: 11.3% > 35: 4%	IOM 2009	8704 children with siblings. 23,264 children with no siblings.	4 4 7 7	Stanford–Binet intelligence scale Graham–Ernhart Block Sort test. Wechsler Intelligence Scale for Children. Wide Range Achievement Test (WRAT): - → Spelling - → Arithmetic	**Maternal’s variables:** age, race, parity, socioeconomic status, and smoking. **Offspring’s variables:** sex and siblings.
Kominiarek et al. 2018 [39]	USA	Cohort	2006–2009	948	27.95 (6.56)	IOM 2009	948	3 5	Differential Ability Scale-II. Wechsler Preschool and Primary Scale of Intelligence.	**Maternal’s variables:** race–ethnicity and education. **Offspring’s variables:** sex.
Li et al. 2018 [15]	China	Cohort	2002–2006	1744	NR	IOM 2009	1307	8.78 (0.82)	Wechsler intelligence scale for children (WISC-IV)	**Maternal’s variables:** parental age, gestational age, county, education, parents’ job, household wealth index, child school level, prenatal micronutrient supplementation, prepregnancy BMI. **Offspring’s variables:** age, sex, older siblings birth weight
Naeye et al. 1981 [12]	USA	Cohort	1959–1966	2183	NA	Own criteria (ii)	2183	0.67 4	Bayley score. IQ.	Stratified by race, gestational age at birth, socioeconomic index (including education, work and family income).
Neggers et al. 2003 [16]	USA	Cohort	1991–1993	355	21.6 (7.3)	IOM 1990	355	5.3 (0.3)	Differential Ability Scales (DAS): - → IQ - → Verbal - → No verbal ability, Peabody gross motor scales (PPVT): - → Reflexes, - → Balance - → Locomotors reflexes. Peabody Picture Vocabulary Test (PPVT): - → Language skills.	**Maternal’s variables:** age, gestational age, mother’s PPVT, tobacco use and maternal alcohol intake and zinc supplementation. **Offspring’s variables**: Offspring’s age, birth weight, home environment and childcare.
Pugh et al. 2015 [40]	USA	Cohort	1983–1986	530	NA	Own criteria (iii)	530	10	Stanford-Binet Intelligence Scale: - → IQ. Wisconsin Card Sorting Test and Trail Making test: - → Cognitive flexibility.	**Maternal’s variables**: race, parity, intelligence, socioeconomic status, education, job, civil status, depressive symptoms and preconception substance use. **Offspring’s variables**: sex and home environment.
Pugh et al. 2016 [35]	USA	Cohort	1983–1986	574	NA	Own criteria (iii)	542	6 and 10 14	Wide Range Achievement Test-Revised (WRAT-R) - → Math. - → Reading. - → Spelling Wechsler Individual Achievement Test (WIAT): - → Math. - → Reading. - → Spelling	**Maternal’s variables:** race, parity, job, intelligence, socioeconomic status, depression and pregestational BMI. **Offspring’s variables**: sex and home environment.
Tanda et al. 2013 [17]	USA	Cohort	1978	3412	25.4 (5)	IOM 2009	3412	5.96 (0.64)	Peabody individual achievement test (PIAT): - → Math. - → Reading.	**Offspring’s variables**: age and siblings.
Tavris et al. 1982 [36]	USA	Cohort	1959–1966	2443	NA	Own criteria (iv)	1638	5	Raven Coloured Progressive” matrices test: - → Intelligence.	Confounding variables without adjusting for them: maternal age, race, parity, maternal education, weight/height ratio, time since the last gestation, parental education and socioeconomic status.
Widen et al. 2018 [37]	USA	Cohort	1959–1966	2084	28.4 (5.9)	NR	1825	9–11	Raven Coloured Progressive” matrices test: - → Intelligence. Peabody Picture Vocabulary Test (PPVT): - → Language skills.	**Maternal’s variables**: race, age, parity, socioeconomic status, education, peabody IQ score and pregestational BMI. **Offspring’s variables**: birth weight and size.

(i) As reported by the original studies in years: intervals, age in whole years at which the measurement was made or average with its standard deviation in years. (ii) Naeye own criteria: suitable GWG 22–32 pounds. (iii) Pugh own criteria 2015 and 2016: suitable GWG was between − 1 to + 1 SD. (iv) Tavris own criteria: suitable GWG 5–29 pounds

*IOM* Institute of Medicine of the United States, *GWG* gestational weight gain, *BMI* body max index, *IQ* intelligence quotient, *NA* not available, *USA* United States of America, *UK* United Kingdom

The included sample size ranged from 355 to 31,968 offspring whose year of birth ranged from 1959 to 2009. The age at which offspring’s cognitive skills were assessed varied from 8 months to 16 years. The average mother’s age ranged from 21.3^9^ to 29.1 years [13].

Seven studies used the IOM recommendations published in 2009 to classify GWG [9, 13, 14, 17, 37–39], one study used those published in 1990 [16], four studies used their own reference values to establish insufficient or excessive GWG [12, 35, 36, 40] and one did not report any classification [15].

### Cognitive skills assessed

The cognitive domains assessed in included studies were: (i) IQ [6, 9, 13, 14, 35, 37, 38], (ii) language-related skills, [13, 14, 16, 17, 35, 37] and (iii) mathematics-related skills [14, 17, 35]. Other cognitive domains included in the studies were: (i) general intelligence [16, 39], (ii) executive function [12, 16, 40], (iii) non-verbal skills [16, 40], and (iv) total scores in academic achievement [38]. Tables S2 to S5 summarize the association between GWG and offspring’s cognitive skills as reported in the original articles and their ES.

The most common variables used in the adjusted analyses were: (i) maternal covariates such as maternal age, pre-pregnancy BMI, parity, race, intelligence, education or gestational age at delivery; (ii) family background covariates including marital status, socioeconomic status or home environment; and (iii) offspring’s covariates such as sex and age. Only two studies did not report adjusted models [12, 36] and three did not report quantitative data of the association between GWG and offspring’s cognitive skills [9, 16]. Among the most important variables that could affect the relationship between GWG and offspring’s cognition between studies included in the meta-analysis: (i) gestational age at birth was taken into account for almost all studies in different ways (only included full-term pregnancies [14, 17, 38–40], adjusted by gestational age [35] or conducted a sensitivity analysis) [13]; (ii) one study only included women with a normal pre-pregnancy BMI [40], and three adjusted by pre-pregnancy BMI [13, 35, 38]; (iii) postnatal environment was taken into account in all studies as different variables.

### Meta-analysis

**Fig. 2:**
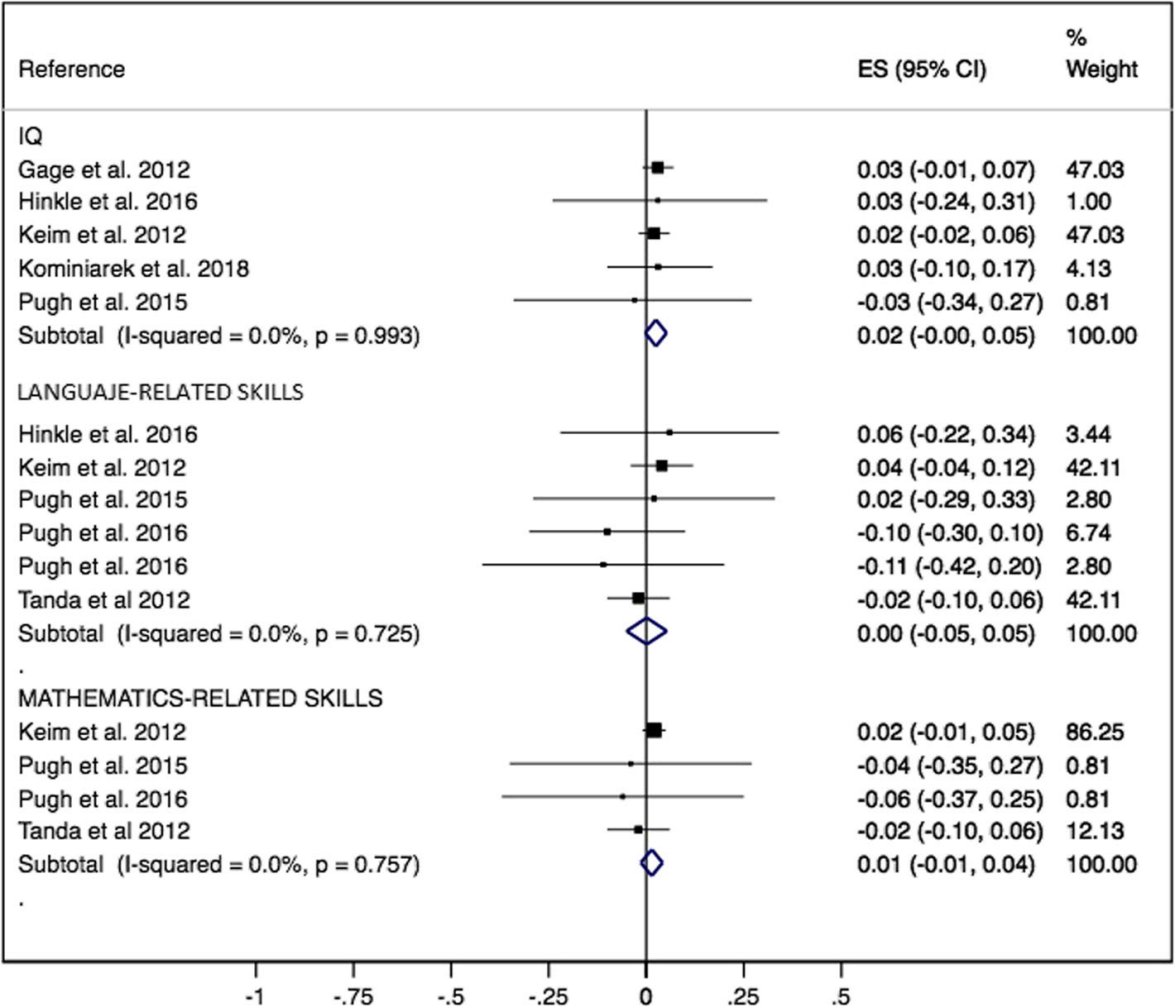
Offspring’s intelligence quotient, language related skills and mathematics related skills forest plot whose mother’s had GWG above recommendations comparing with GWG within recommendations

The ES for the association of GWG above recommendations with offspring’s IQ was 0.02 (95% CI -0.00, 0.05; I^2^ = 0.00%), language related skills 0.00 (95% CI -0.05, 0.05; I^2^ = 0.00%) and mathematics related skills 0.01 (95% CI -0.01, 0.04; I^2^ = 0.00%) (Fig. 2).

**Fig. 3:**
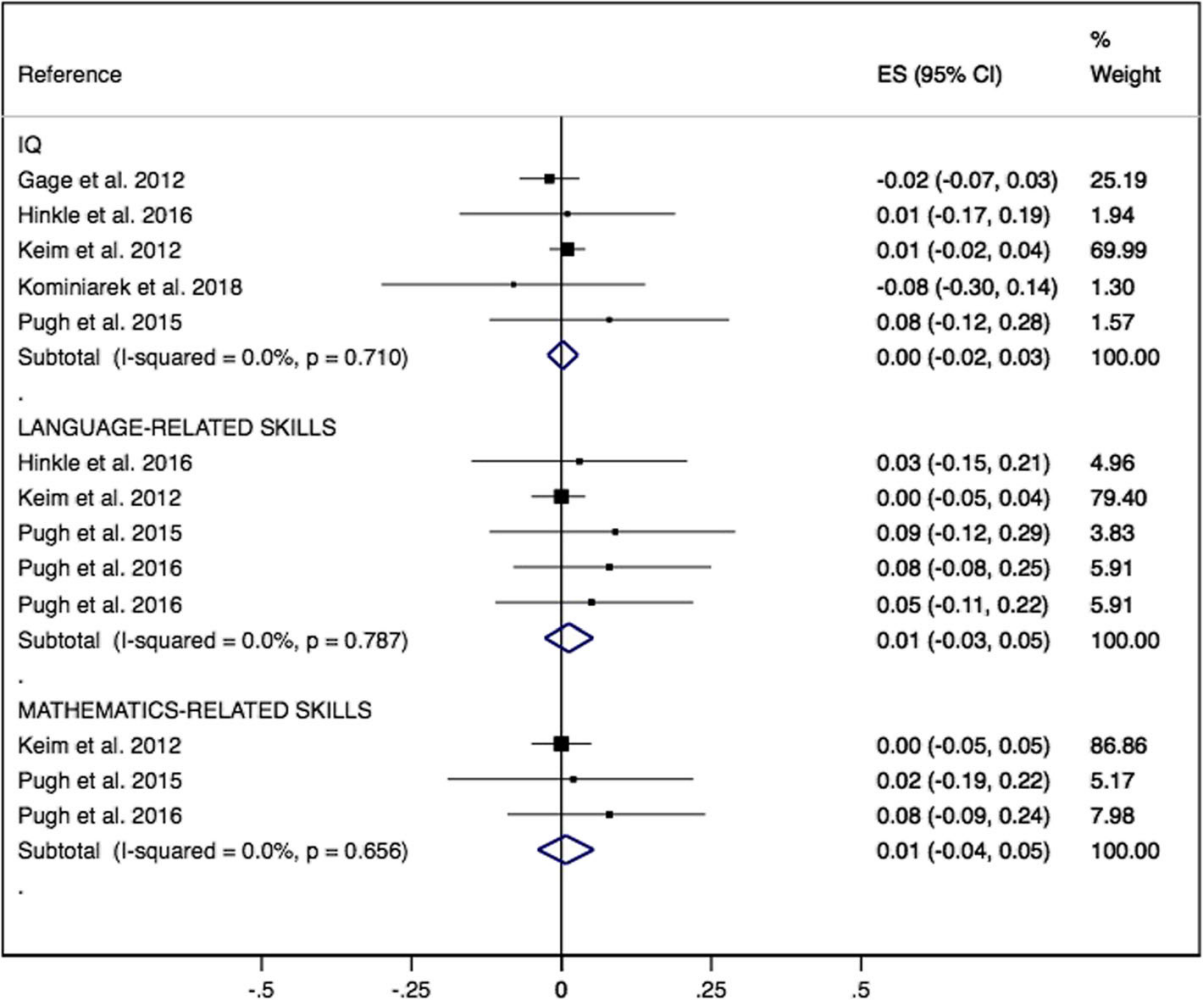
Offspring’s intelligence quotient, language related skills and mathematics related skills forest plot whose mother’s had GWG below recommendations comparing with GWG within recommendations

Furthermore, the ES for the association of GWG below recommendations with offspring’s IQ was 0.00 (95% CI -0.02, 0.03; I^2^ = 0.00%), language-related skills 0.02 (95% CI -0.03, 0.05; I^2^ = 0.00%) and mathematics-related skills 0.01 (95% CI: − 0.04, 0.05; I^2^ = 0.00%) (Fig. 3).

Other cognitive domains were not included in the meta-analysis, but these studies did not find association between GWG and general intelligence [16, 39], executive functions [12, 16, 39, 40] non-verbal skills [16, 40] or short term memory [40]. However, GWG was positively associated with better academic achievement at 16 years of age [38].

#### Subgroup analyses

We did not observe differences by GWG classification or age when we conducted subgroup analyses (Tables S6 to S17).

#### Sensitivity analyses.

Sensitivity analyses suggested that the pooled ES or the heterogeneity were not modified in any model after removing the included studies one by one (Tables S18 to S23).

#### Assessment of publication Bias.

Funnel plots appear symmetrical and the Egger test revealed that there was no evidence of publication bias in any model (*p* values higher than 0.1), except for the model comparing offspring’s language related skills between mothers who had GWG below recommendation versus mothers who had GWG within recommendations (*p* = 0.02).

### Quality of reporting

The level of compliance for the risk of bias assessment varied from 55 to 91% (Table S24). None of the studies justified the number of participants. Furthermore, four studies reported a quantitative statement of consenters or non-consenters [13, 16, 35, 40] and four reported reasons for loss to follow up [15, 17, 36, 40]. Regarding statistical analysis methods, one study took into account loss to follow up [13] and two considered missing data in the analysis [13, 40]. Finally, one reported the impact of biases estimated quantitatively [17].

## Discussion

The findings of this systematic review and meta-analysis support that the weight gain during pregnancy above recommendations could have a small positive influence in offspring’s IQ, but the expected relationship between GWG above recommendations and language or mathematics related skills was not confirmed. Finally, GWG below the recommendations was not associated neither with IQ, nor with language and mathematics related skills.

Our pooled estimates found a small positive relationship between GWG and offspring’s IQ, although not statistically significant, in line with a previous study that reported a positive linear association between GWG until 28 weeks and offspring’s IQ [38]. This fact could be explained because greater GWG may produce greater maternal fat deposition, and might result in a greater delivery of glucose and fatty acids, which could positively influence the fetal brain development [38]. The results of the included studies are inconsistent, while some studies reported association between GWG and offspring’s cognitive skills [9, 14, 38], others did not provide any association [12–17, 35, 37, 40]. To explain these mixed results, previous published articles reported biological mechanisms that could underlying the potential relationships. For example, an increased inflammatory markers level as a result of excessive maternal fat deposition, which might contribute to an adverse environment for fetal brain development [41] and epigenetic alterations [42–44]. In contrast, synapse formation and myelination are vulnerable to undernutrition, hence insufficient GWG could be associated with suboptimal neurodevelopment [45]. All studies reported weak associations between GWG and offspring’s cognitive skills and this fact could be due to the exposure of GWG is limited by pregnancy period and offspring’s cognitive skills are influenced by other postnatal factors that could dilute the effect of perinatal factors in older offspring such as home environment or physical activity during childhood [46, 47]. The magnitude of the association between GWG and offspring’s cognitive skills is similar to those reported for other exposures around pregnancy and lactation periods, such as pre-pregnancy BMI or breast feeding [48, 49].

Several reasons can be adduced to explain variability across studies and differences between adjusted and unadjusted models found in included studies [13, 38] may be due to the variability in the covariates included in the models. Obesity before pregnancy have a negative influence on offspring’s cognitive skills [48] and it is possible that GWG within recommendations might mitigate this negative association [9], although the pathways behind this relationship are not entirely clear [38, 41, 42, 45]. Not only pre-pregnancy obesity, but also other perinatal and postnatal exposures could in greater or lesser extend to influence cognitive development [47, 50–52]. Thus, two studies proposed a novel analysis by siblings to control for observed variables or unobserved factors that are shared by siblings, because they account for all genetic and environmental factors in common [9, 14].

### Strengths and limitations

The large total sample size accumulated by included studies, the lack of publication bias (except for the model that compared offspring’s language related skills between mothers who had GWG below recommendation versus mothers who had GWG within recommendations.), the standardized tests used to assess cognitive skills were according to offspring’s age and the consistency of results proven in sensitivity analyses are among the strengths of this study.

However, some limitations should also be acknowledged in this article: (i) the offspring’s cognitive measuring tools were different in each study; (ii) some studies included subsamples of disadvantaged subgroups or population subgroups, which could influence the magnitude of the associations; (iii) cohorts were living in countries where the health related behaviours are presumably different, which could influence cognitive development, although this could be mitigate in the adjusted model [9, 14, 16, 36]; (iv) we could not performed a subgroup analyses by pre-pregnancy BMI, gestational age at birth, offspring’s age at cognitive assessment or trimester specific because there were few studies that reported these subgroup analyses; (v) studies included used different criteria for GWG classification; (vi) the lack of trials make impossible to stablish causality between GWG and offspring’s cognition.

### Implication for research

The GWG should be appropriately manage by clinicians for improving neonatal and maternal outcomes, among them offspring’s cognitive skills. Thus, prenatal care providers, who are in a privileged position to manage GWG, should give individualized advice on physical activity and diet to women to prioritize an adequate or a careful excessive GWG because it could improve offspring’s IQ according to our findings [53–57].

Due to the variability reported by original articles and the differences in sample characteristics across studies and study designs we highlight in the importance considerations for future research: (i) the use of 2009 IOM’s guidelines to classify GWG, (ii) the use of validated tests to assess cognitive skills and (iii) if sibling models are not possible, the control of potential confounders in the analysis [9, 14].

## Conclusion

Our systematic review and meta-analysis supports that GWG above recommendations could improve offspring’s IQ. However, we found no association between GWG and offspring’s cognitive skills, although differences in the sample characteristics and analyses may explain the variability in results reported by the original articles. The generalizability of our results could be limited due to the lack of homogeneous design of included studies, but our findings could provide an initial approach for elucidating the association between GWG and offspring’s cognition. Potential influence of perinatal and postnatal variables could be behind this inconsistence. Further high-quality studies are needed including population-based samples, using the same GWG classification criteria and validated offspring’s cognitive assessment tools.

## Supplementary Information

**Additional file 1:** **Table S1.** Search strategy for MEDLINE database.

**Additional file 2:** **Tables S2 to S5.** Calculations.

**Additional file 3:** **Tables S6 to S17.** Subgroup analyses by GWG classification. **Tables S18 to S23**. Sensitivity analyses.

**Additional file 4:** **Table S24.** Quality assessment by Quality of reporting of observational longitudinal research scale.

## Data Availability

Supporting data can be obtained from the corresponding author.

## Abbreviations

GWG: Gestational weight gain
IOM: Institute of Medicine
BMI: Body mass index
ES: Effect size
IQ: Intelligence quotient
