# Supplementary material for: Gestational weight gain and offspring’s cognitive skills: a systematic review and meta-analysis
Source: BMC Pediatr. 2020 Nov 26;20:533. doi: 10.1186/s12887-020-02429-7 (PMC7690030; doi:10.1186/s12887-020-02429-7)
Supplement: Supplementary file 1 — Additional file 1: Table S1. Search strategy for MEDLINE database. [file 12887_2020_2429_MOESM1_ESM.docx]

# Table S1. Search strategy for MEDLINE.

| Gestational  OR  Pregnancy  OR  Maternal  OR  Antepartum  OR  Prenatal | AND | “weight gain”  OR  “weight change”  OR  “obesity | AND | “academic achievement"  OR  "academic grades"  OR  "academic behavior"  OR  "academic performance"  OR  "academic"  OR  "attention"  OR  "classroom behavior"  OR  "cognition"  OR  "cognitive development"  OR  "cognitive function"  OR  "cognitive control"  OR  "cognitive achievement"  OR  "executive"  OR  "executive function"  OR  "intellectual"  OR  "intelligence"  OR  "neurodevelopment"  OR  "memory"  OR  "metacognition"  OR  “vocabulary” | AND | "birth"  OR  "infant"  OR  “child”  OR  "childhood"  OR  "children"  OR  "offspring"  OR "adolescence" |
| --- | --- | --- | --- | --- | --- | --- |
