# Supplementary material for: Gestational weight gain and offspring’s cognitive skills: a systematic review and meta-analysis
Source: BMC Pediatr. 2020 Nov 26;20:533. doi: 10.1186/s12887-020-02429-7 (PMC7690030; doi:10.1186/s12887-020-02429-7)
Supplement: Supplementary file 2 — Additional file 2: Tables S2 to S5. Calculations. [file 12887_2020_2429_MOESM2_ESM.docx]

**Table S2. Offspring’s cognition reported as means difference (CI).**

| **Study** | **Tool for assessment offspring’s cognition** | **GWG above recomendations** | **Suitable GWG** | **GWG below recommendations** | **ES model 1** | **ES model 2** |
| --- | --- | --- | --- | --- | --- | --- |
| Gage et al. (2012) | - School entry assessment score. - Wechsler Intelligence Scale for Children:   - Standarized IQ | 0.011 (- 0.045, 0.067)  0.066 (-0.002, 0.131) | As reference  As reference | -0.075 (-0.127, 0.023)  -0.03 (-0.089, 0.029) | **0.01 (-0.05, 0.06)**  **0.03 (-0.01, 0.07)** | **-0.04 (-0.09, 0.01)**  **-0.02 (-0.07, 0.03)** |

Gestational weight gain (GWG); not available (NA); effect size (ES).

Model 1: Offspring’s cognitive skills of mother’s that GWG was above recommendations versus offspring’s cognitive skills of mother’s that GWG was between recommendations.

Model 2: Offspring’s cognitive skills of mother’s that GWG was below recommendations versus offspring’s cognitive skills of mother’s that GWG was between recommendations.

**Table S3. Offspring’s cognition reported as standardized β (CI).**

| **Study** | **Tool for assessment offspring’s cognition** | **GWG above recomendation** | **Suitable GWG** | **GWG below recommendations** | **ES model 1** | **ES model 2** |
| --- | --- | --- | --- | --- | --- | --- |
| Keim et al. (2012) | - Stanford–Binet IQ intelligence scale - Wechsler Intelligence Scale for Children. - Wide Range Achievement Test (WRAT)   - Spelling  - Arithmetic | 0.55 (-0.93, 2.04)  0.85 (-0.41, 2.11)  1.04 (-0.46, 2.54)  1.41 (0.13, 2.70)  0.45 (-0.66, 1.55) | As reference  As reference  As reference  As reference  As reference | 0.26 (-1.20, 0.67)  0.52 (-0.27, 1.31)  0.15 (-0.79, 1.09)  -0.11 (-0.92, 0.69)  0.03 (-0.67, 0.72) | 0.01 (-0.06, 0.09)  0.02 (-0.03, 0.07)  0.03 (-0.05, 0.10)  0.04 (-0.04, 0.11)  0.02 (-0.01, 0.05) | 0.01 (-0.04, 0.05)  0.01 (-0.03, 0.06)  0.00 (-0.04, 0.05)  -0.00 (-0.05, 0.04)  0.0 (-0.05, 0.05) |

Gestational weight gain (GWG); not available (NA); effect size (ES).

Model 1: Offspring’s cognitive skills of mother’s that GWG was above recommendations versus offspring’s cognitive skills of mother’s that GWG was between recommendations.

Model 2: Offspring’s cognitive skills of mother’s that GWG was below recommendations versus offspring’s cognitive skills of mother’s that GWG was between recommendations.

**Table S4. Offspring’s cognition reported as unstandardized B (CI).**

| **Study** | **Tool for assessment offspring’s cognition** | **GWG above recomendations** | **Suitable GWG** | **GWG below recommendations** | **ES model 1** | **ES model 2** |
| --- | --- | --- | --- | --- | --- | --- |
| Hinkle et al. (2016) | - Wechsler Preschoolers and Primary Scales of Intelligence (WPPSI-R), - Full Scale IQ:   2º trimester  3er trimester  **Pooled ES**   - Verbal IQ: | 3.2 (-2.8, 9.2)  -0.7 (-8.2, 6.9) | As reference  As reference | 0.2 (-3.2, 3.6)  0.2 (-3.6, 3.9) | 0.10 (-0.34, 0.54)  -0.01 (-0.38, 0.35)  **0.03 (-0.24, 0.31)** | 0.01 (-0.26, 0.27)    0.01 (-0.25, 0.26)  **0.01 (-0.17, 0.19**) |
|  | 2º trimester  3er trimester  **Pooled ES**   - Performance IQ   2º trimester  3er trimester  **Pooled ES** | 3.5 (-2.4, 9.5)  1.2 (-6.2. 8.7)  1.8 (-4.7, 8.2)  -3.4 (-11.5, 4.7) | As reference  As reference  As reference  As reference | 0.8 (-2.6, 4.1)  1 (-2.6, 4.7)  -0.5 (-4.2, 3.1)  -1.3 (-5.2, 2.7) | 0.11 (-0.33, 0.56)  0.02 (-0.34, 0.39)  **0.06 (-0.22, 0.34)**  0.05 (-0.39, 0.5)  -0.06 (-0.43, 0.3)  **-0.02 (-0.3, 0.26)** | 0.03 (-0.24, 0.29)  0.03 (-0.22, 0.29)  **0.03 (-0.15, 0.21)**  -0.02 (-0.28, 0.25)  -0.04 (-0.3, 0.21)  **-0.03 (-0.21, 0.15)** |

Gestational weight gain (GWG); not available (NA); effect size (ES).

Model 1: Offspring’s cognitive skills of mother’s that GWG was above recommendations versus offspring’s cognitive skills of mother’s that GWG was between recommendations.

Model 2: Offspring’s cognitive skills of mother’s that GWG was below recommendations versus offspring’s cognitive skills of mother’s that GWG was between recommendations.

| **Study** | **Tool for assessment offspring’s cognition** | **GWG above recomendations** | **Suitable GWG** | **GWG below recommendations** | **ES model 1** | **ES model 2** |
| --- | --- | --- | --- | --- | --- | --- |
| Pugh et al. (2015) | - Stanford-Binet Intelligence Scale: - IQ. - Visual. - Verbal. - Quantitative. - Short term memory. - Wisconsin Card Sorting Test - Part B, Trail Making test | -1.1 (-4.2, 3.7)  -1.9 (-5.8, 1.9)  0.8 (-2.8, 4.4)  -1.4 (-5.0, 2.4)  -1.2 (-5.4, 2.9)  0.94 (0.82, 1.1)  15 (1.8, 28) | 0  0  0  0  0  0  0 | 1.7 (-0.27, 3.7)  1.3 (-1.1, 3.8)  2.2 (-0.1, 4.5)  0.6 (-1.8, 3.0)  1.7 (-0.9, 4.4)  1.1 (0.96,1.2)  -0.33 (-0.88,8.1) | -0.33 (-0.34, 0.27)  -0.05 (-0.36, 0.26)  0.02 (-0.29, 0.33)  -0.04 (-0.35, 0.27)  -0.03 (-0.34, 0.28)  -0.17 (-0.48, 0.14)  0.11 (-0.2, 0.42) | 0.08 (-0.12, 0.28)  0.05 (-0.15, 0.25)  0.09 (-0.12, 0.29)  0.02 (-0.19, 0.22)  0.06 (-0.14, 0.26)  0.02 (-0.18, 0.2)  0.00 (-0.2, 0.2) |
| Pugh et al. (2016) | - Wide Range Achievement Test-Revised (WRAT-R) Wechsler Individual Achievement Test (WIAT): - Math - Reading - Spelling | -2.47 (-6.1, 1.1)  -3.75 (-7.1, -0.4)  -3.90 (-7.8, -0.2) | As reference  As reference  As reference | 1.77 (-0.3, 3.8)  2.01 (-0.2, 4.2)  1.33 (-1.0, 3.3) | -0.06 (-0.37, 0.25)  -0.11 (-0.42, 0.20)  -0.10 (-0.30, 0.10) | 0.08 (-0.09, 0.24)  0.08 (-0.08, 0.25)  0.05 (-0.11, 0.22) |
| Tanda et al (2012) | - Peabody individual achievement test (PIAT): - Mathematics scores - Reading scores | -0.48 (0.50)  -0.53 (0.49) | As reference  As reference | NA  NA | -0.02 (-0.10, 0.06)  -0.02 (-0.10, 0.06) |  |

**Table S4. Adjusted model: Offspring’s cognition reported as unstandardized B (CI).**

Gestational weight gain (GWG); not available (NA); effect size (ES).

Model 1: Offspring’s cognitive skills of mother’s that GWG was above recommendations versus offspring’s cognitive skills of mother’s that GWG was between recommendations.

Model 2: Offspring’s cognitive skills of mother’s that GWG was below recommendations versus offspring’s cognitive skills of mother’s that GWG was between recommendations.

**Table S5. Adjusted model: Offspring’s cognition reported as means (SD).**

| **Study** | **Tool for assessment offspring’s cognition** | **GWG above recomendations** | **Suitable GWG** | **GWG below recommendations** | **ES model 1** | **ES model 2** |
| --- | --- | --- | --- | --- | --- | --- |
| Kominiarek et al. (2018) | - Wechsler Preschool and Primary Scale of Intelligence. | 95.5 (15.4) | As reference | 93.9 (13.6) | **0.03 (-0.1, 0.17)** | **-0.08 (-0.3, 0.14)** |

Standard deviation (SD); Gestational weight gain (GWG); not available (NA); effect size (ES).

Model 1: Offspring’s cognitive skills of mother’s that GWG was above recommendations versus offspring’s cognitive skills of mother’s that GWG was between recommendations.

Model 2: Offspring’s cognitive skills of mother’s that GWG was below recommendations versus offspring’s cognitive skills of mother’s that GWG was between recommendations.
