# Supplementary material for: Gestational weight gain and offspring’s cognitive skills: a systematic review and meta-analysis
Source: BMC Pediatr. 2020 Nov 26;20:533. doi: 10.1186/s12887-020-02429-7 (PMC7690030; doi:10.1186/s12887-020-02429-7)
Supplement: Supplementary file 3 — Additional file 3: Tables S6 to S17. Subgroup analyses by GWG classification. Tables S18 to S23. Sensitivity analyses. [file 12887_2020_2429_MOESM3_ESM.docx]

**Table S6: Subgroup analysis of GWG above recommendations versus GWG within recommendations and offspring’s intelligence quotient model by GWG classification.**

| Classification criteria | Number of studies | ES (95% CI) | I^2^ | p |
| --- | --- | --- | --- | --- |
| 2009 IOM’s classification | 4 | 0.03 (-0.00, 0.05) | 0.00 | 0.99 |
| Other | 1 | -0.03 (-0.33, 0.27) | 0.00 | 1 |
| OVERALL | 5 | 0.02 (-0.00, 0.05) | 0.00 | 0.99 |

ES: Effect size; CI (Confidence Interval).

**Table S7: Subgroup analysis of GWG below recommendations versus GWG within recommendations and offspring’s intelligence quotient model by GWG classification.**

| Classification criteria | Number of studies | ES (95% CI) | I^2^ | p |
| --- | --- | --- | --- | --- |
| 2009 IOM’s classification | 4 | 0.00 (-0.02, 0.03) | 0.00 | 0.67 |
| Other | 1 | 0.08 (-0.12, 0.28) | 0.00 | 1 |
| OVERALL | 5 | 0.00 (-0.02, 0.03) | 0.00 | 0.71 |

ES: Effect size; CI (Confidence Interval).

**Table S8: Subgroup analysis of GWG above recommendations versus GWG within recommendations and offspring’s language-related skills model by GWG classification.**

| Classification criteria | Number of studies | ES (95% CI) | I^2^ | p |
| --- | --- | --- | --- | --- |
| 2009 IOM’s classification | 3 | 0.01 (-0.04, 0.07) | 0.00 | 0.55 |
| Other | 3 | -0.08 (-0.22, 0.07) | 0.00 | 0.79 |
| OVERALL | 6 | 0.00 (-0.05, 0.05) | 0.00 | 0.73 |

**Table S9: Subgroup analysis of GWG below recommendations versus GWG within recommendations and offspring’s language-related skills model by GWG classification.**

| Classification criteria | Number of studies | ES (95% CI) | I^2^ | p |
| --- | --- | --- | --- | --- |
| 2009 IOM’s classification | 2 | 0.00 (-0.05, 0.05) | 0.00 | 0.75 |
| Other | 3 | 0.07 (-0.11, 0.29) | 0.00 | 0.95 |
| OVERALL | 5 | 0.01 (-0.03, 0.05) | 0.00 | 0.79 |

ES: Effect size; CI (Confidence Interval).

**Table S10: Subgroup analysis of GWG above recommendations versus GWG within recommendations and offspring’s mathematics-related skills model by GWG classification.**

| Classification criteria | Number of studies | ES (95% CI) | I^2^ | p |
| --- | --- | --- | --- | --- |
| 2009 IOM’s classification | 2 | 0.02 (-0.01, 0.05) | 0.00 | 0.71 |
| Other | 2 | -0.02 (-0.10, 0.06) | 0.00 | 0.81 |
| OVERALL | 4 | 0.01 (-0.01, 0.04) | 0.00 | 0.75 |

**Table S11: Subgroup analysis of GWG below recommendations versus GWG within recommendations and offspring’s mathematics-related skills model by GWG classification.**

| Classification criteria | Number of studies | ES (95% CI) | I^2^ | p |
| --- | --- | --- | --- | --- |
| 2009 IOM’s classification | 2 | 0.00 (-0.05, 0.05) | 0.00 | 1 |
| Other | 3 | 0.06 (-0.07, 0.19) | 0.00 | 0.65 |
| OVERALL | 3 | 0.01 (-0.04, 0.05) | 0.00 | 0.66 |

ES: Effect size; CI (Confidence Interval).

**Table S12: Subgroup analysis of GWG above recommendations versus GWG within recommendations and offspring’s IQ model by schooling.**

| Classification criteria | Number of studies | ES (95% CI) | I^2^ | p |
| --- | --- | --- | --- | --- |
| Pre-school children | 3 | 0.01 (-0.02, 0.04) | 0.00 | 0.38 |
| School children | 2 | 0.03 (-0.01, 0.07) | 0.00 | 0.96 |
| OVERALL | 5 | 0.02 (-0.00, 0.05) | 0.00 | 0.67 |

**Table S13: Subgroup analysis of GWG above recommendations versus GWG within recommendations and offspring’s mathematics-related skills model by schooling.**

| Classification criteria | Number of studies | ES (95% CI) | I^2^ | p |
| --- | --- | --- | --- | --- |
| Pre-school children | 3 | -0.01 (-0.09, 0.06) | 0.00 | 0.59 |
| School children | 2 | 0.01 (-0.06, 0.08) | 0.00 | 0.52 |
| OVERALL | 5 | 0.00 (-0.05, 0.05) | 0.00 | 0.72 |

**Table S14: Subgroup analysis of GWG above recommendations versus GWG within recommendations and offspring’s language-related skills model by schooling.**

| Classification criteria | Number of studies | ES (95% CI) | I^2^ | p |
| --- | --- | --- | --- | --- |
| Pre-school children | 1 | -0.02 (-0.1, 0.06) | 0.00 | 1 |
| School children | 3 | 0.02 (-0,01,0,04) | 0.00 | 0.82 |
| OVERALL | 4 | 0,01 (-0,01, 0,04) | 0.00 | 0.76 |

**Table S15: Subgroup analysis of GWG below recommendations versus GWG within recommendations and offspring’s IQ model by schooling.**

| Classification criteria | Number of studies | ES (95% CI) | I^2^ | p |
| --- | --- | --- | --- | --- |
| Pre-school children | 3 | 0,01 (-0,02, 0.04) | 0.00 | 0.73 |
| School children | 2 | -0.02 (-0.06, 0.03) | 0.00 | 0.34 |
| OVERALL | 5 | 0.02(-0.02, 0.03) | 0.00 | 0.71 |

**Table S16: Subgroup analysis of GWG below recommendations versus GWG within recommendations and offspring’s mathematics-related skills model by schooling.**

| Classification criteria | Number of studies | ES (95% CI) | I^2^ | p |
| --- | --- | --- | --- | --- |
| Pre-school children | 2 | 0.04 (-0.08, 0.16) | 0.00 | 0.87 |
| School children | 3 | 0.01 (-0.04, 0.06) | 0.00 | 0.49 |
| OVERALL | 5 | 0.01 (-0.03, 0.05) | 0.00 | 78.7 |

**Table S17: Subgroup analysis of GWG below recommendations versus GWG within recommendations and offspring’s language-related skills model by schooling.**

| Classification criteria | Number of studies | ES (95% CI) | I^2^ | p |
| --- | --- | --- | --- | --- |
| Pre-school children | 0 | - | - | - |
| School children | 3 | 0.01 (-0.04, 0.05) | 0.00 | 0.79 |
| OVERALL | 3 | 0.01 (-0.04, 0.05) | 0.00 | 0.79 |

**Table S18.** **Sensitivity analysis of GWG above recommendations versus GWG within recommendations and offspring’s intelligence quotient model.**

| **Study removed** | **OR** | **Low limit** | **Upper Limit** | **I^2^** | **p** |
| --- | --- | --- | --- | --- | --- |
| **Gage et al. 2012** | 0,02 | -0,02 | 0,06 | 0,00 | 0,99 |
| **Hinkle et al. 2016** | 0,03 | -0,00 | 0,05 | 0,00 | 0,97 |
| **Keim et al. 2012** | 0,03 | -0,01 | 0,07 | 0,00 | 0,99 |
| **Kominiarek et al. 2018** | 0,03 | -0,00 | 0,05 | 0,00 | 0,97 |
| **Pugh et al. 2015** | 0,03 | -0,00 | 0,05 | 0,00 | 0,99 |

**Table S19. Sensitivity analysis of GWG below recommendations versus GWG within recommendations and offspring’s intelligence quotient model.**

| **Study removed** | **OR** | **Low limit** | **Upper Limit** | **I^2^** | **p** |
| --- | --- | --- | --- | --- | --- |
| **Gage et al. 2012** | 0,01 | -0,02 | 0,04 | 0,00 | 0,77 |
| **Hinkle et al. 2016** | 0,02 | -0,02 | 0,03 | 0,00 | 0,54 |
| **Keim et al. 2012** | -0,02 | -0,06 | 0,03 | 0,00 | 0,72 |
| **Kominiarek et al. 2018** | 0,00 | -0,02 | 0,03 | 0,00 | 0,66 |
| **Pugh et al. 2015** | 0,00 | -0,02 | 0,03 | 0,00 | 0,67 |

**Table S20. Sensitivity analysis of GWG above recommendations versus GWG within recommendations and offspring’s language related skills.**

| **Study removed** | **OR** | **Low limit** | **Upper Limit** | **I^2^** | **p** |
| --- | --- | --- | --- | --- | --- |
| Hinkle et al. 2016 | -0,00 | -0,05 | 0,05 | 0,00 | 0,62 |
| Keim et al. 2012 | -0,03 | -0,10 | 0,04 | 0,00 | 0,86 |
| Pugh et al. 2015 | 0,00 | -0,05 | 0,05 | 0,00 | 0,59 |
| Pugh et al. 2016 (a) | 0,01 | -0,04 | 0,06 | 0,00 | 0,78 |
| Pugh et al. 2016 (b) | 0,00 | -0,05 | 0,06 | 0,00 | 0,67 |
| Tanda et al. 2012 | 0,02 | -0,05 | 0,09 | 0,00 | 0,67 |

**Table S21. Sensitivity analysis of GWG below recommendations versus GWG within recommendations and offspring’s language related skills.**

| **Study removed** | **OR** | **Low limit** | **Upper Limit** | **I^2^** | **p** |
| --- | --- | --- | --- | --- | --- |
| Hinkle et al. 2016 | 0,01 | -0,03 | 0,06 | 0,00 | 0,65 |
| Keim et al. 2012 | 0,06 | -0,03 | 0,15 | 0,00 | 0,97 |
| Pugh et al. 2015 | 0,01 | -0,03 | 0,06 | 0,00 | 0,77 |
| Pugh et al. 2016 | 0,01 | -0,04 | 0,06 | 0,00 | 0,80 |
| Pugh et al. 2016 | 0,01 | -0,03 | 0,06 | 0,00 | 0,69 |

**Table S22. Sensitivity analysis of GWG above recommendations versus GWG within recommendations and offspring’s mathematics related skills.**

| **Study removed** | **OR** | **Low limit** | **Upper Limit** | **I^2^** | **p** |
| --- | --- | --- | --- | --- | --- |
| Keim et al. 2012 | -0,02 | -0,10 | 0,05 | 0,00 | 0,97 |
| Pugh et al. 2015 | 0,01 | -0,01 | -0,04 | 0,00 | 0,59 |
| Pugh et al. 2016 | 0,02 | -0,01 | 0,04 | 0,00 | 0,62 |
| Tanda et al. 2012 | 0,02 | -0,01 | 0,05 | 0,00 | 0,80 |

**Table S23. Sensitivity analysis of GWG below recommendations versus GWG within recommendations and offspring’s mathematics related skills.**

| **Study removed** | **OR** | **Low limit** | **Upper Limit** | **I^2^** | **p** |
| --- | --- | --- | --- | --- | --- |
| Keim et al. 2012 | 0,06 | -0,07 | 0,19 | 0,00 | 0,65 |
| Pugh et al. 2015 | 0,01 | -0,04 | 0,06 | 0,00 | 0,36 |
| Pugh et al. 2016 | 0,00 | -0,05 | 0,05 | 0,00 | 0,85 |
